# The challenge of ionisation chamber dosimetry in ultra-short pulsed high dose-rate Very High Energy Electron beams

**DOI:** 10.1038/s41598-020-65819-y

**Published:** 2020-06-03

**Authors:** M. McManus, F. Romano, N. D. Lee, W. Farabolini, A. Gilardi, G. Royle, H. Palmans, A. Subiel

**Affiliations:** 1 Medical Radiation Science, National Physical Laboratory, Hampton Road, Teddington, Middlesex TW11 0LW United Kingdom; 2 Medical Physics and Biomedical Engineering, University College London, Gower Street, London, WC1E 6BT United Kingdom; 3 MedAustron, Marie Curie-Strasse 5, 2700 Wiener Neustadt, Austria; 4 CERN, Geneva, 1217 Switzerland; 5 Istituto Nazionale di Fisica Nucleare, Sezione di Catania, Catania, Via S Sofia 64, I-95123 Catania, Italy; 6 CEA-Saclay, IRFU, 91191 Gif-sur-Yvette, France

**Keywords:** Radiotherapy, Radiotherapy

## Abstract

High dose-rate radiotherapy, known as FLASH, has been shown to increase the differential response between healthy and tumour tissue. Moreover, Very High Energy Electrons (VHEEs) provide more favourable dose distributions than conventional radiotherapy electron and photon beams. Plane-parallel ionisation chambers are the recommended secondary standard systems for clinical reference dosimetry of electrons, therefore chamber response to these high energy and high dose-per-pulse beams must be well understood. Graphite calorimetry, the UK primary standard, has been employed to measure the dose delivered from a 200 MeV pulsed electron beam. This was compared to the charge measurements of a plane-parallel ionisation chamber to determine the absolute collection efficiency and infer the ion recombination factor. The dose-per-pulse measured by the calorimeter ranged between 0.03 Gy/pulse and 5.26 Gy/pulse, corresponding to collection efficiencies between 97% and 4%, respectively. Multiple recombination models currently available have been compared with experimental results. This work is directly applicable to the development of standard dosimetry protocols for VHEE radiotherapy, FLASH radiotherapy and other high dose-rate modalities. However, the use of secondary standard ionisation chambers for the dosimetry of high dose-per-pulse VHEEs has been shown to require large corrections for charge collection inefficiency.

## Introduction

The radiation oncology community is constantly exploring possibilities to increase the efficacy of radiotherapy treatments by increasing the therapeutic window between tumour control probability (TCP) and normal tissue complication probability (NTCP). Technological developments and innovations in radiation treatment delivery and patient imaging allow for more accurate tumour targeting whilst minimizing the damage to the surrounding healthy tissues. However, these ongoing advances generate relatively slow improvements in radiotherapy outcomes.

Ultra-high dose-rate irradiations, known as FLASH radiotherapy, rely on delivery of therapeutic doses at instantaneous dose-rates over four orders of magnitude higher than those currently used in conventional radiotherapy. Such an extremely short delivery of radiation leads to remarkable reduction of normal tissue toxicity with respect to conventional dose-rate radiotherapy. These effects were reported five decades ago^1–4^, however translation to the clinical practice was not pursued due to lack of availability of clinically suitable radiation sources. The recent years have stimulated the revival of FLASH following the report from the Franco-Swiss team in the mouse model^5^, which has been continued in the subsequent investigations^6–10^. Moreover, 2019 has seen the first patient treated with FLASH radiotherapy using a 5.6 MeV electron beam^11^. It is worth noting that FLASH is a biological effect and not defined by the beam which is responsible for inducing the biological response. However, it is common to specify beam parameters that may cause the FLASH effect. All of the radiation response studies published so far indicate the robustness of the FLASH effect as it has been studied across various animal models and numerous organs^12^. Most of these studies have been conducted with ultra-high dose-rate electron beams generated by a linear accelerator with energies up-to 20 MeV. Such a low energy of the electron beam generates a major obstacle in their translation to future clinical trials of FLASH due to limited penetration depth. The application of Very High Energy Electrons (VHEEs) in radiotherapy, with energies up-to 250 MeV, could overcome this depth limitation due to significantly increased practical range and improved penumbra for deep-seated tumours with respect to currently available clinical photon beams, which (in contrast to VHEE) cannot be delivered in a regime which induces the FLASH effect. Moreover, VHEE beams can provide more conformal dose distributions to deep seated tumours, in comparison to current advanced electron radiotherapy techniques, whilst reducing the integral dose and organ-at-risk dose^13–15^. There is also the possibility of focusing VHEE beams into the patient, reducing peak surface and exit doses for a single beam by more than one order of magnitude compared with a collimated beam^16^. Moreover, VHEE radiotherapy would benefit from reduced scattering and divergence, leading to a reduction in healthy tissue irradiation surrounding the tumour. Currently there are no radiobiological data available evaluating efficacy of VHEEs for radiotherapy applications. However, there are a number of research facilities^17–20^ enabling access to these beams, paving the way for development of radiobiological and pre-clinical programs for VHEEs and their applicability to FLASH. Recently, a possible clinical VHEE delivery system is being developed by a team at the SLAC National Accelerator Laboratory, known as “PHASER”^21^. Future generations of this system will be designed to deliver electron beams from a number of LINAC structures surrounding the patient. This removes the need for complex moving parts such as a gantry and collimators as well as reducing the treatment costs compared to proton and ion therapy. This system would also provide fast enough delivery to achieve the dose-rate requirements of FLASH radiotherapy.

Laser-driven beams can deliver instantaneous dose-rates which are several orders of magnitude larger than both conventional therapy and FLASH techniques^22^. This type of beam delivery could prove to be more cost-effective and compact than a standard LINAC structure^23^. Moreover, laser-driven accelerators can deliver high energy radiation and therefore could provide both high dose-rate delivery and VHEE beams.

One of the challenges that needs to be addressed prior to the translation of FLASH and VHEE studies into the clinical stage is the development of accurate dosimetry protocols and characterization of suitable detectors that could serve as secondary standard dosimeters in hospitals for this radiotherapy regime. Dosimetry at high dose-rate is notoriously complicated and it is of the utmost importance to understand the effects that will influence detector response. Dosimetric measurements in the first FLASH patient treatment with electrons were obtained using radiochromic films and alanine, both of which require post-irradiation processing and are not practical for regular use in clinical practice. Previous work has shown that ionization chambers, used routinely in radiation therapy as secondary standard dosimeters, exhibit significant recombination effects with increased dose-rates^24–28^, however no systematic study is available for VHEE beams.

Plane parallel type ionisation chambers are the recommended dosimeter for reference dosimetry in clinical electron beams^29^. The absorbed dose-to-water, ${D}_{w,Q}$, is obtained from an ionisation chamber measurement as:

1 $${D}_{w,Q}=M{k}_{s}{k}_{pol}{k}_{TP}{k}_{Q,{Q}_{0}}{N}_{D,w,{Q}_{0}}$$

where $M$ is the charge reading of the chamber, ${k}_{s}$ is the recombination correction factor, ${k}_{pol}$ is the polarity correction factor, ${k}_{TP}$ is the temperature and pressure correction factor, ${k}_{Q,{Q}_{0}}$ is the beam quality correction factor, applied when the user operates in a beam quality, $Q$, different from that of the reference quality, ${Q}_{0}$, and ${N}_{D,w,{Q}_{0}}$ is the calibration coefficient of the ionisation chamber in the reference beam quality. For high dose-per-pulse beams, the ion recombination effect is expected to be large due to the high charge density in each electron pulse. This work aims to determine the relationship between the recombination factor of an ionisation chamber and a wide range of dose-per-pulse values for an electron LINAC with higher instantaneous dose-rates (dose-rate in a pulse) than any previous dosimetric studies. The absolute dose-per-pulse was measured with a National Physical Laboratory (NPL) designed graphite calorimeter (similar to that described by *Duane et al*.^30^) and compared to dose obtained from the PTW Roos ionisation chamber. As the calorimeter measurement system is dose-rate independent, it was possible to determine the recombination factor of the Roos chamber for various collecting voltages. Moreover, the calorimeter does not require either a calibration or any post-irradiation processing. This type of absolute recombination determination has not been previously attempted. This study is a systematic investigation exploring the feasibility of employing currently available ionization chambers for future FLASH and VHEE radiotherapy over a wide range of high doses-per-pulse values. The recommended recombination correction procedures are valid in the near saturation region of the ion chamber, where the chamber is capable of collecting nearly all of the produced charge^29^. Analytical recombination models developed by *Boag et al*.^31^ have been studied thoroughly at clinical dose-rates, however their applicability to the Roos chamber in the high dose-per-pulse regime of this work has not been investigated before. In the low charge collection efficiency region of the chamber, these models are expected to be invalid and as such, high dose-per-pulse specific analytical recombination models have been developed by *Di Martino et al*. *(2005)* for Intraoperative Radiotherapy (IORT) applications^27^. The validity of both analytical models, proposed by Boag and Di Martino, were tested in high dose-per-pulse VHEEs. Additionally, a logistic model for the recombination in high dose-per-pulse beams, used by *Petersson et al*.^26^, has also been investigated. This manuscript provides a groundwork for future developments of ion chamber-based dosimetry of FLASH and ultra-high dose-per-pulse VHEE radiotherapy.

## Results

Beam output values of a number of radiotherapy techniques from published studies are compared to this study in Table 1. FLASH was seen to operate at the highest dose-rate of up-to 117 Gy/s. The instantaneous dose-rate of the beam in this study was several orders of magnitude larger than all but laser-driven beams. This is due to the the short pulse structure of the beam, described in the Beam characteristics section.

**Table 1:** Beam dose-rate, instantaneous dose-rate and dose-per-pulse in this study are compared to specific referenced examples from several radiotherapy techniques.

	Dose-Rate (Gy/s)	Instantaneous Dose-Rate (Gy/s)	Dose-Per-Pulse (Gy)
Conventional^38^	2.4 × 10^−2^–2.4 × 10^−1^	10^2^–10^3^	10^−4^–10^−3^
IORT^27^	10^−2^–10^−1^	≤10^4^	10^−2^–10^−1^
FLASH^5^	0.03–117	6 × 10^3^–5 × 10^6^	6 × 10^−3^–5
Laser-Driven^22^	10–10^2^	≤2.4 × 10^9^	2 × 10^−3^–3.2 × 10^−3^
This Study	0.17–50.41	5.0 × 10^6^–3.1 × 10^8^	0.03–5.26

The dose-to-water conversion factor, ${C}_{g,w}$, used to convert from the measured dose-to-graphite from the calorimeter into the required dose-to-water, was calculated to be 1.0912 with statistical uncertainty of ±0.001%, as described in Section 4.3. The subsequent dose-per-pulse to water is displayed in Table 2.

**Table 2:** Dose-per-pulse and *k*_*s*,*abs*_ values, calculated using Eq. (3), corresponding to each charge-per-pulse in the beam.

Nominal Beam Charge (nC/pulse)	*D*_*w*,*cal*_ (Gy/pulse)	*k*_*s*,*abs*_				*k*_*s*,*TVA*_			
		75 V	200 V	350 V	600 V	75 V	200 V	350 V	600 V
0.05	0.03	1.30	0.98	0.89	0.78	1.65	1.24	1.13	0.99
0.20	0.20	3.41	1.87	1.56	1.14	3.57	1.96	1.63	1.20
0.25	0.14	2.46	1.33	2.05	No Data	3.73	2.02	3.12	No Data
1.00	0.67	6.00	3.07	2.12	1.58	4.60	2.35	1.63	1.21
2.20	1.25	8.80	4.12	2.80	1.94	6.65	3.12	2.12	1.47
3.00	1.95	11.96	5.67	No Data	2.58	6.30	2.99	No Data	1.36
4.50	2.63	14.99	6.87	4.59	3.07	7.49	3.43	2.29	1.54
6.00	3.66	18.94	8.54	5.63	3.81	8.24	3.72	2.45	1.66
7.50	4.12	19.54	8.77	5.69	3.74	8.46	3.80	2.46	1.62
9.00	4.56	21.38	9.30	5.99	4.23	10.39	4.52	2.91	2.06
10.50	5.26	22.99	9.95	6.50	4.24	10.83	4.69	3.06	2.00
11.00	5.04	21.81	9.46	6.00	3.84	10.63	4.61	2.92	1.87

*k*_*s*,*abs*_ values are seen to decrease with increasing voltage, as expected. *k*_*s*,*abs*_ refers to the absolute recombination factor calculated from calorimeter measurements and *k*_*s*,*TVA*_ refers to the recombination factor calculated using the graphical TVA method.

**Figure 1:**
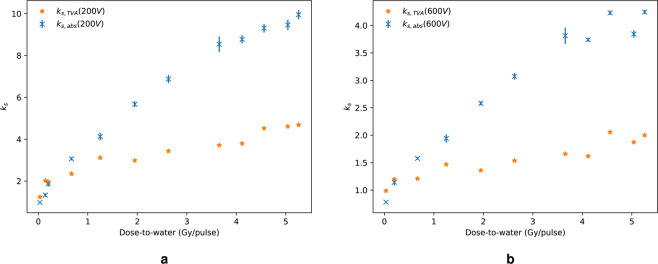
*k*_*s*,*abs*_ values from dose-to-calorimeter and dose-to-chamber ratio (crosses) compared with ${k}_{s,TVA}$ calculated using the graphical TVA method from Jaffé plots (stars). The linear region of the Jaffé plot was taken to be between 75 V and 200 V, with ${k}_{s}$ for 200 V (**a**) and 600 V (**b**).

The recombination factor, as a function of the dose-per-pulse measured with the calorimeter, for collecting voltages of 200 V and 600 V, is shown in Fig. 1a and 1b, respectively. At the highest dose-per-pulse of $5.26\pm 0.04$ Gy/pulse, the absolute recombination factor, ${k}_{s,abs}$, was calculated to be $9.95\pm 0.13$ and $4.24\pm 0.05$ for 200 V and 600 V, respectively. These values are compared to the Two-Voltage Analysis (TVA) method, ${k}_{s,TVA}$, calculated from linear fits to Jaffé plot data, which is described in the Two-voltage method section. The recombination factors determined from absolute measurements, ${k}_{s,abs}$, and determined from Jaffé plots, ${k}_{s,TVA}$, are displayed in Table 2. The nominal beam charge values were determined using an integrated current transformer installed along the beam and represent the total charge of each electron pulse. In general, there was a close to linear relationship between the nominal charge-per-pulse delivered by the beam and the dose-per-pulse measured by the calorimeter. At a 200 V collecting voltage, ${k}_{s,abs}$ and ${k}_{s,TVA}$ are in general agreement below approximately 0.5 Gy/pulse. However, a larger discrepancy is seen when using 600 V. Above 1 Gy/pulse, ${k}_{s,TVA}$ diverges from the absolute value, significantly underestimating the recombination factor. The uncertainties on ${k}_{s,abs}$ reported here account for a type-A standard uncertainty evaluation of both the calorimeter and ion chamber measurements as well as the type-B uncertainty evaluation of the dose-to-graphite to dose-to-water conversion factor and calibration coefficient, with a coverage factor of $k=1$, as described in the Monte Carlo calculations and Calculation of recombination factor sections, respectively. The combined standard uncertainty in the dose-per-pulse to water, which includes the type-A uncertainty of dose-to-graphite from the calorimeter and the type-B uncertainty of the dose-to-water conversion, ranged between 0.78% and 5.80%, depending on the charge-per-pulse delivered. This variation in uncertainty is likely due to user systematic errors, as the beam had to be manually switched on and off during calorimeter exposure, as well as fluctuations in mean charge output and beam spread, which varied with the number of bunches-per-pulse. The Roos chamber standard uncertainty, including type-A measurement uncertainty evaluation and the uncertainty associated with the calibration coefficient, was also subject to some larger variations, particularly at lower collecting voltages, and was observed to rise up-to approximately 6%. This, again, is likely due to the chosen beam parameters, leading to larger fluctuations in charge output of the beam. The vast majority of uncertainties, however, remained within 1% to 1.2%. For simplicity at this preliminary stage, the underlying contributions to the total uncertainties in dose quoted here, such as the gap and fluence correction factors for the calorimeter and perturbation correction factors for the the Roos chamber, have not been separately included.

**Figure 2:**
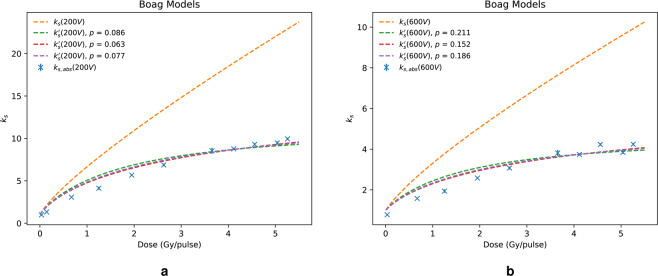
Comparison between the Boag model prediction of ${k}_{s,abs}$ and that calculated from the calorimeter/chamber dose ratio at 200 V (**a**) and 600 V (**b**).

The Boag models, described in the Boag model section, are compared with data at 200 V and 600 V in Fig. 2a and b, respectively. It is evident that the original Boag model, Eq. (4), is invalid in the high dose-per-pulse regime, overestimating significantly the recombination factor and providing an almost linear relationship between recombination and dose-per-pulse. However, the three free-electron fraction (FEF) models, given by Eqs. (5), (6) and (7), fit reasonably well to the data using a least-squares optimisation of the FEF, $p$. This is the fraction of charge collected by the chamber which originates purely from the collection of free-electrons instead of negative ions. It was found to increase with increasing collecting voltage and Eq. (7) was shown to be close to the average of Eqs. (5) and (6) both of which agree with the findings of *Boag et al*.^31^. Moreover, Boag found that for a collecting voltage of 600 V, $p$ was approximately 0.2. This is in very good agreement with the estimated value calculated in this work which was equal to 0.211 using Eq. (5).

**Figure 3:**
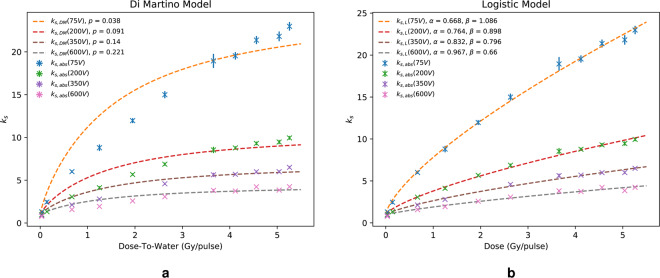
Plot (**a**) shows the Di Martino model, ${k}_{s,DM}$, fitted to data, whilst plot (**b**) shows the logistic model fit, ${k}_{s,L}$.

Fig. 3a shows data fitted with the model of *Di Martino et al*.^27^. As the Di Martino model is based upon Eq. (5), it exhibits similar behaviour and similar estimations of $p$ when compared to the Boag models. For example, a least-squares optimisation of $p$ yielded $p=0.221$ and $p=0.211$ at 600 V for the Di Martino (Eq. (9)) and Boag model (Eq. (5)), respectively. Table 3 shows the calculated $p$ values for the Boag and Di Martino models at each investigated collecting voltage along with their respective uncertainties. The Di Martino model shows general qualitative agreement over the whole dose range for all voltages investigated.

**Table 3:** Estimated *p* values of the Boag and Di Martino models alongside their associated uncertainties, with *k* = 1 coverage factor, determined by taking the square root of the diagonal product of the covariance matrix corresponding to the least squares estimation.

Collecting Voltage (V)	Boag ${k{\prime} }_{s}$		Di Martino *k*_*s*,*DM*_		Boag ${k{\prime\prime} }_{s}$		Boag ${k{\prime} {\prime} {\prime} }_{s}$	
	$p$	Uncert.	$p$	Uncert.	$p$	Uncert.	$p$	Uncert.
75	0.036	0.002	0.038	0.003	0.027	0.001	0.032	0.002
200	0.086	0.005	0.091	0.006	0.063	0.003	0.077	0.004
350	0.121	0.018	0.140	0.009	0.086	0.016	0.106	0.018
600	0.211	0.014	0.221	0.015	0.152	0.009	0.186	0.011

Fig. 3b shows the logistic model, described by *Petersson et al*.^26^. This model has two fitting constants, $\alpha$ and $\beta$, as defined in Eq. (10). It is immediately clear that the logistic model fits very well over the whole dose range. The fitting constants were again estimated using a least squares optimisation and are displayed alongside their uncertainties in Table 4.

**Table 4:** Logistic model fitting constants, *α* and *β*, estimated using least squares optimisation for each collecting voltage, with corresponding uncertainties at *k* = 1 coverage factor.

Collecting Voltage (V)	Logistic *k*_*s*,*L*_			
	*α*	*β*	Uncert. (*α*)	Uncert. (*β*)
75	0.668	1.086	0.068	0.100
200	0.764	0.898	0.092	0.095
350	0.832	0.796	0.141	0.113
600	0.967	0.660	0.120	0.110

**Figure 4:**
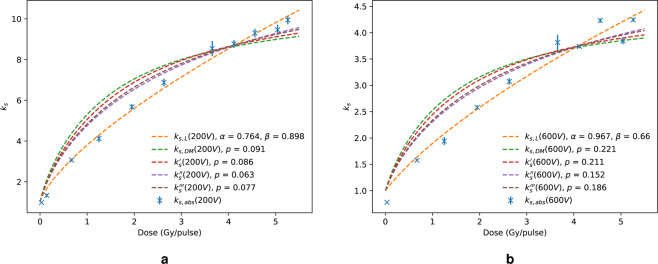
All models compared for 200 V (**a**) and 600 V (**b**) collecting voltage.

All models are compared at 200 V and 600 V in Fig. 4a and b, respectively. There was minimal variation in ${k}_{s,abs}$ estimation at each voltage between the Boag FEF models and the Di Martino model, which gave the worst fit to data at both 200 V and 600 V. Moreover, the accuracy of the logistic model is evident from Fig. 4 in comparison to the FEF based models.

## Discussion and Conclusions

Unique measurements using both a graphite calorimeter and Roos ionisation chamber were used to determine the absolute recombination factor ${k}_{s,abs}$ of the ion chamber as a function of dose-per-pulse when exposed to high dose-per-pulse VHEEs. The recombination factor was calculated for chamber collecting voltages of 75 V, 200 V, 350 V and 600 V. The value of ${k}_{s}$ was found to increase over the investigated dose range for each collecting voltage. The largest recombination factor was found to be approximately ${k}_{s,abs}\approx 24$ at a collecting voltage of 75 V and a dose-per-pulse of 5.26 Gy/pulse, corresponding to a chamber collection efficiency of $\mathrm{4 \% }$ ($\mathrm{1/}{k}_{s,abs}$). The highest collection efficiency of 97%, measured at 0.03 Gy/pulse, is similar to what would be expected in a standard clinical radiotherapy beam. This significant decrease in collection efficiency indicates that understanding the process of ion collection in the chamber will be fundamental in the translation of FLASH therapy and laser-driven sources into clinical practice.

Three values in Table 2 show that ${k}_{s,abs}$ was measured to be less than 1 for ${D}_{w,cal}=0.03$ and collecting voltages of $200$ V, 350 V and 600 V. This corresponds to a collection efficiency greater than 100%, which is not physical. This is likely the result of both an uncertainty on the value of ${k}_{Q,{Q}_{0}}$ and charge multiplication effects at high voltages, where the ions are provided enough energy by the electric field that they themselves can further ionise the gas within the cavity. This leads to an increase in the charge collected by the chamber which did not originate from the initial electron pulse, therefore overestimating the dose delivered. Table 2 shows that, for a nominal beam charge of 0.25 nC/pulse, the measured ${D}_{w,cal}$ and corresponding ${k}_{s,abs}$ were lower than what was measured for 0.2 nC/pulse at 75 V and 200 V. At 0.25 nC/pulse, only two bunches were sent in each pulse, with a charge-per-bunch of 0.125 nC. This low number of bunches can cause an instability in the total charge-per-pulse output of the beam, as the mechanism by which the bunches are selected is not $\mathrm{100 \% }$ accurate. Due to the high charge-per-bunch, any fluctuations in the average number of bunches transported could result in a significant reduction or increase of the measured dose from the calorimeter. With a larger number of bunches-per-pulse, the average charge-per-pulse is distributed between more bunches, therefore the average charge output will remain more stable. Similar arguments can be made to describe the lower ${D}_{w,cal}$ and ${k}_{s,abs}$ values found at 11 nC/pulse in comparison to 10.5 nC/pulse.

Several models were compared with ${k}_{s,abs}$, including the alternative graphical method of the TVA, described in the Two-voltage method section. It was found that the TVA method diverged from the absolute recombination factor above approximately $0.5$ Gy/pulse. Moreover, this method significantly underestimates ${k}_{s,abs}$ at the highest investigated dose-per-pulse by up-to $\mathrm{70 \% }$. The standard Boag model, Eq. (4), is recommended by the IPEM Code of Practice for electron dosimetry^32^. This model is reported to only be accurate in the near saturation region i.e. when the recombination factor is small. Eq. (4) was found to significantly overestimate ${k}_{s,abs}$ by more than a factor of $2$ at the highest measured dose-per-pulse. Additional models compared were that of Boag and Di Martino, which included the free-electron fraction, $p$. These models gave reasonable qualitative fits to the data, however did not accurately predict the recombination factor over the whole dose-per-pulse range. The FEF was estimated by Eqs. (5), (6), (7) and (9) for the three Boag models and the Di Martino model, respectively. As the Di Martino model is based upon Eq. (5), they estimate similar $p$ values at all voltages, for example, 0.221 and 0.211 at 600 V, respectively. Di Martino investigated a dose-per-pulse ranging between 3.97 and 12.36 cGy/pulse, resulting in a ${k}_{s}$ range between 1.58 and 2.34 at 100 V^27^. The $p$ value estimated here at 75 V using the Di Martino model was 0.036, significantly lower than that determined in Di Martino’s work, who found $p=0.179$ at 100 V. The $p$ values estimated in this work, using both the Boag and Di Martino models, are in close agreement with that stated by *Boag et al*.^31^, who found approximately $p=0.2$ at 600 V. However, the Boag experiment was carried out for a chamber exposed to short-pulsed x-rays and with an electrode spacing of 0.31 cm, 1 mm wider than the Roos chamber used in this work. Variations in the cavity size and geometry may attribute to differences in the measured free electron fraction. Eq. (7) gave a $p$ value which was close to the average of Eqs. (5) and (6), as previously found by Boag^31^. As expected, $p$ was observed to increase with increasing collecting voltage. Finally, the logistic model, proposed by *Petersson et al*. *(2017)*, provided the most accurate prediction across the full dose-per-pulse range studied in this work. They investigated the recombination in the Advanced Markus chamber^26^, which has a smaller cavity volume with respect to the Roos, therefore the fitting constants estimated in this work cannot be compared directly.

Absolute dosimeters such as the graphite calorimeter are complex devices and typically their operation is restricted to primary standards laboratories. Therefore, in practice, the recombination factor will be determined through only relative methods. The FEF and logistic fitting constants estimated here could be used to determine the recombination factor of the Roos chamber in high dose-per-pulse VHEE beams without the need for direct comparison with absolute dose measurements. However, it is clear that the use of extremely high dose-per-pulse beams have a significant impact on the secondary standard dosimeters which are used daily in clinics. In particular, this work provides an insight into the affect that $p$ has on the overall recombination factor, a phenomenon in which very little experimental data exists.

As shown in Table 1, the instantaneous dose-rate per-pulse in this work is significantly larger than that of IORT, for which ample data is already available, or FLASH techniques. The dose-per-pulse values, on the other hand, are of similar orders of magnitude as FLASH and IORT. The results presented here reflect an extreme and unique pulse structure and it is likely that it is the dose and instantaneous dose-rate per-pulse which have a large effect on the measured recombination factor and not the overall dose-rate of the beam. The high instantaneous dose-rate and low repetition rates measured in this work make it an ideal comparison for laser-plasma studies. However, reports of the instability of the laser-plasma source and broad energy spectra suggest there is more work to be done before an accurate and comprehensive dosimetric study can be conducted. This work can likely benefit further developments in laser-driven proton and electron dosimetry, as the instantaneous dose-rates are of similar magnitudes^33^. Recombination factors determined here depict the most extreme scenario when employing ultra-short pulsed high dose-rate radiation therapy.

Certain irradiation parameters need to be controlled in order to enhance the FLASH effect in mammalian tissue. When looking closer at the temporal dosimetry characteristics of the available *in vivo* studies demonstrating the FLASH effect, there is evidence that beam parameters such as dose-per-pulse, instantaneous dose-rate and the number of pulses delivered can considerably impact FLASH radiotherapy outcomes^5–9,34^. The radiobiological data available so far on the FLASH effect indicates that better normal tissues sparing is obtained by delivering a lower number of pulses and, hence, higher dose-per-pulse, which would decrease collection efficiency in ionization chambers. More radiation research is needed to define the most optimal beam parameters maximizing enhancement of normal tissue sparing. However, it is clear that a rigorous and accurate system to monitor dose needs to be developed for clinical use to allow for the safe delivery of ultra-high dose-per-pulse radiotherapy. Measurements here show that the absolute recombination factor in a Roos ionisation chamber is strongly dependent on the dose-per-pulse and leads to significant correction factors. A possible solution is the use of different ionisation chamber geometries with smaller electrode spacing or a cylindrical cavity shape. This would result in an increased electric field strength at lower voltages and therefore help collect charge more effectively, reducing recombination. This study provides a groundwork on which a wide variety of further dosimetric studies in high dose-per-pulse radiation including laser-plasma accelerators, VHEEs and electron and proton FLASH radiotherapy can be conducted.

## Materials and Methods

### Detectors and setup

**Figure 5:**
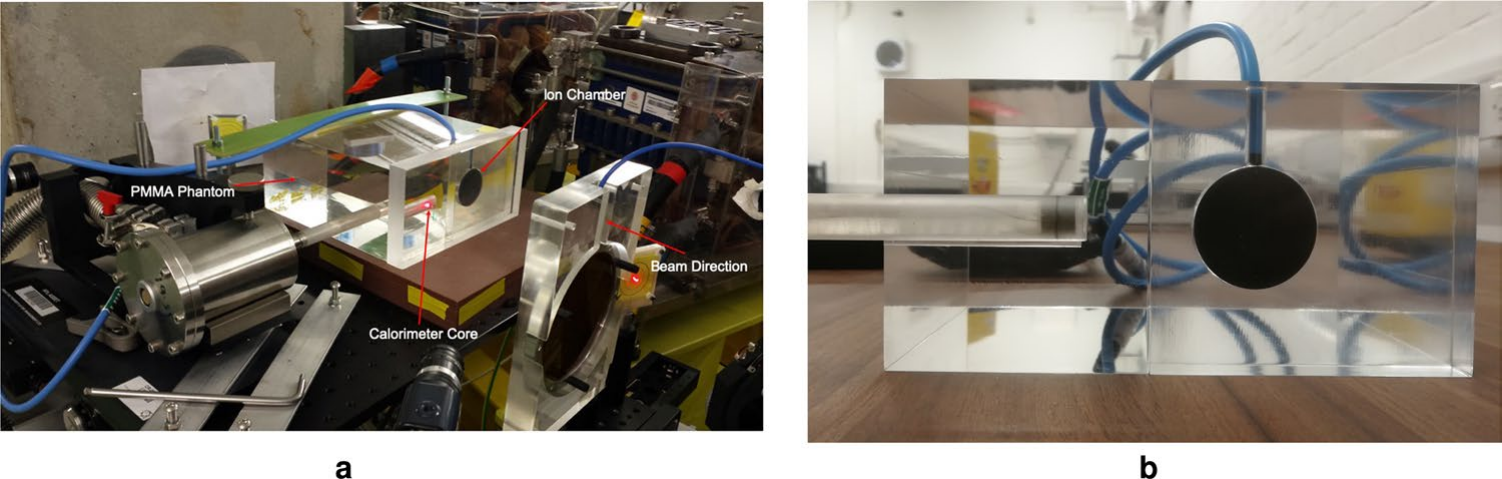
(**a**) The test-stand at the CLEAR facility, with the calorimeter, ion chamber and monitor chamber placed along the beam line with the beam travelling from right to left. The calorimeter core can be seen at the end of the PMMA sleeve (**b**). This shows the front surface of the custom PMMA phantom with Roos chamber insert placed to the right of the calorimeter sleeve. The PMMA build-up blocks, used to ensure the reference depth of the detectors was at 8 cm, are not included in the photographs.

The reference ionisation chamber used throughout was the PTW Roos Type-34001 (SN: 002496). The effective point of measurement of the chamber was placed at approximately 8 cm water equivalent depth in a custom build poly-methyl methacrylate (PMMA) phantom with dimensions $10\times 20\times 20\,{{\rm{cm}}}^{3}$. Adjacent to the ion chamber was an NPL graphite calorimeter positioned with the centre of the graphite core at the same water equivalent depth in the PMMA phantom and a 5 cm separation from the centre of the ion chamber, perpendicular to the beam direction. The phantom setup can be seen in Fig. 5a and b, below. The calorimeter core is a cylindrical piece of graphite measuring 7 mm in both height and diameter. It is housed in a graphite jacket of 1 mm thickness, with a 1 mm vacuum gap between the jacket and the core. This is then placed at the end of a 163.5 mm long hollow cylindrical PMMA sleeve with wall thickness of 1.05 mm. The centre of the calorimeter core is placed 1 cm from the tip of the PMMA sleeve, while the opposite end is attached to a vacuum pump. The whole setup was placed on a remotely movable stand such that each detector could be moved into the beam for individual measurements without stopping the beam. Thermistors embedded in the core are used to measure the temperature rise caused by the ionising radiation to the order of mK. LabView software developed at the NPL was used to record the temperature rise in the core and calculate the absorbed dose for a number of identical runs.

### Beam characteristics

The calorimeter and chamber were exposed to a quasi-monoenergetic pulsed electron beam of approximately 200 MeV at the CLEAR user facility at CERN^17^. The energy spread of the beam was measured between $\mathrm{0.25 \% }$ and $\mathrm{6.5 \% }$, depending on the optimisation of beam parameters. Each electron pulse was formed of a variable number of shorter electron bunches, with an adjustable charge-per-bunch range between 0.001 nC to 1.5 nC. Each bunch has a length of approximately 1 ps. A collection of electron bunches is defined as a pulse train, therefore dose measurements throughout this work are referred to in dose-per-pulse. The bunch spacing was 666 ps, therefore the length of the pulse train was dependent on the number of bunches. For example, if one required 150 bunches-per-pulse, the pulse length would be approximately 100 ns. By adjusting the charge-per-bunch and the number of bunches-per-pulse, it was possible to accurately investigate charge-per-pulse values ranging from 0.05 nC to 11 nC. The collection time of the ion chamber is of the order of *μ*s, therefore the chamber can be assumed to only be sensitive to the total macro pulse and not the individual bunches. The maximum standard deviation of beam charge was measured to be approximately $0.78$ nC, for a charge-per-pulse of $10.5$ nC. A $0.4$ mm thick silicon scattering foil was used to increase the field size of the circular beam to approximately 5 mm *σ* in both the $x$ and $y$ directions at the surface of the phantom and was continuously measured using a YAG:Ce crystal scintillator screen, with thickness of $0.5$ mm, placed along the beamline. The beam size varied by approximately $1.5$ mm over the course of the experimental campaign. The beam was then scattered to a sufficient size by the phantom, ensuring that the beam would fully cover both the ion chamber cavity and the calorimeter core, with cross sectional areas of 191 mm^2^ and 49 mm^2^, respectively, at the reference depth of the detectors. A correction factor for the difference in sensitive volume size between the detectors was not accounted for in this work.

A PTW Monitor Type-7862 (SN: 000696) chamber was placed between the vacuum window and the phantom setup allowing for the normalisation of any beam fluctuations in the measurements, however was not used in the determination of ${k}_{s,abs}$ as it was also subject to recombination effects at high dose-per-pulse. The Roos chamber measured charge at collecting voltages of $75$ V, $200$ V, $350$ V and $600$ V, while the monitor chamber was kept constant at its nominal operating voltage of $400$ V. Due to space limitations, the front face of the setup was placed at $50$ cm from the vacuum exit window of the beamline which comprised of a $0.1$ mm thick aluminium foil.

### Monte Carlo calculations

Monte Carlo (MC) simulations in GEANT4 10.05, with the QBBC physics list, were used to estimate the reference depth at which the chamber would be placed in the phantom^35^. The MC setup consisted of a circular beam, reproducing the experimental lateral beam dimensions, positioned $50$ cm from the surface of the PMMA phantom in air, with a $\mathrm{0.5 \% }$ Gaussian energy spread at full width half maximum. The dose was scored as a function of depth along the central axis of the phantom with a scoring volume of $5\times 5\times 200\,{{\rm{mm}}}^{3}$, split into 400 voxels along the $z$ axis, providing a resolution of $0.5$ mm. As no reference dosimetry protocols exist for VHEEs, the standard clinical recommendations of the IPEM electron dosimetry Code of Practice (CoP) were followed and the reference depth was determined to be approximately $8$ cm, marginally further than the depth of maximum dose^32^. Build-up blocks of $7$ cm thickness were placed at the surface of the phantom such that the chamber was at this depth.

The NPL calorimeter measures dose-to-graphite, ${D}_{g,cal}$, therefore MC simulations were used to calculate the equivalent dose-to-water according to ${D}_{w,cal}={C}_{g,w}{D}_{g,cal}$, where ${C}_{g,w}$ is the graphite to water dose conversion factor.

### Calculation of recombination factor

The Roos chamber calibration coefficient for $200$ MeV was unknown. Therefore, for indicative purposes, the calibration coefficient was determined for the Roos chamber by taking measurements at $12$ MeV in the clinical electron LINAC at the NPL. This reading was then cross calibrated against a secondary standard Roos chamber and scaled to $200$ MeV using the following equation.

2 $${N}_{D,w,{Q}_{0}}{k}_{Q,{Q}_{0}}={N}_{D,w,{Q}_{0}}\frac{{({W}_{air}/e)}_{Q}}{{({W}_{air}/e)}_{{Q}_{0}}}\frac{{p}_{Q}}{{p}_{{Q}_{0}}}\frac{{s}_{water,air,Q}}{{s}_{water,air,{Q}_{0}}}$$

where the subscripts, $Q$ and ${Q}_{0}$, indicate the electron beam qualities of $200$ MeV and $12$ MeV, respectively, ${({W}_{air}/e)}_{i}$ is the average energy required to produce an ion pair, ${p}_{i}$ is the ion chamber perturbation factor and ${s}_{water,air,i}$ is the stopping power ratio of water to air, at beam quality $i$ where $i=Q,{Q}_{0}$. This equation assumes no variation on the mean energy required to produce an ion pair and also that the ion chamber perturbation factor is unity and has no variation with increasing energy^29^. The calibration coefficient was for the Roos chamber at $200$ MeV was therefore only dependant on the stopping power ratio and was calculated to be $7.74\times {10}^{7}\pm 7.74\times {10}^{5}$ Gy/C ($k=1$) in water. In this proof-of-principle work, ${k}_{pol}$ was also considered to be unity and not taken into account as this factor is primarily influenced by scattered electrons and electrons which stop in the chamber, both of which can be considered small at $200$ MeV.

The absolute value of the recombination factor was calculated from the ratio of the absolute dose determined from the calorimeter and the dose calculated from the ion chamber using Eq. 1.

3 $${k}_{s,abs}=\frac{{D}_{w,cal}}{M{k}_{pol}{k}_{TP}{N}_{D,w,{Q}_{0}}{k}_{Q,{Q}_{0}}}$$

where ${D}_{w,cal}$ is the dose-to-water measured from the calorimeter.

### Ion recombination

#### Boag model

Several Boag recombination models have been compared. The original model, Eq. (4), which Boag presented in 1950^36^. The remaining three, Eqs. (5–7) are given in Boag’s later work published in 1996^31^. These three models take into account the fraction of charge measured by the chamber which is a result of the collection of free electrons before they attached to neutral oxygen molecules. The chamber collection efficiencies were calculated using the following equations.

4 $$\frac{1}{{k}_{s}}=f=\frac{1}{u}ln\mathrm{(1}+\mathrm{u)}$$

5 $$\frac{1}{{k{\prime} }_{s}}=f{\prime} =\frac{1}{u}ln\left(1+\frac{{e}^{pu}-1}{p}\right)$$

6 $$\frac{1}{{k{\rm{{\prime} }}{\rm{{\prime} }}}_{s}}=f{\rm{{\prime} }}{\rm{{\prime} }}=p+\frac{1}{u}ln(1+(1-p)u)$$

7 $$\frac{1}{{k{\prime} {\prime} {\prime} }_{s}}=f{\prime} {\prime} {\prime} =\lambda +\frac{1}{u}ln\left(1+\frac{{e}^{\lambda \mathrm{(1}-\lambda )u}-1}{\lambda }\right)$$

with

$$u=\frac{\mu d{s}^{2}}{V}$$

where each equation represents the collection efficiency, $f$, of the ionisation chamber at a particular collecting voltage $V$, $p$ is the free-electron fraction, $\lambda =1-\sqrt{1-p}$, $\mu$ is a constant equal to $10.2\times {10}^{8}$ Vm^−2^ Gy^−1^ and is related to the recombination coefficient and the ion mobilities, $d$ is the dose-per-pulse and $s$ is the electrode spacing in the chamber.  Eq. (4) calculates solely the collection efficiency of negative oxygen ions in the chamber, assuming that all free electrons in the chamber, immediately after the radiation pulse, become attached to neutral oxygen molecules. Eqs. (5), (6) and (7) however, incorporate $p$ into the equation of the collection efficiency following different approximations of the distributions of negative charge in the chamber cavity.

#### Two-voltage method

In the pulsed beam case, one can assume a linear relationship between 1/*V* and 1/*M*, where *M* is the measured charge in the chamber. An alternative approach to the TVA method^29^, similar to that used by *DeBlois et al*. *(1999)*, can also be performed graphically using Jaffé plots of 1/*V* against 1/*M*^37^. In practice, these plots only show linearity at low collecting voltages. Fitting a straight line between 1/*M*_1_ and 1/*M*_2_, where *M*_1_ and *M*_2_ represent the charge collected at voltages ${V}_{1}$ and ${V}_{2}$, respectively, in the linear region. Extrapolating to infinite voltage, one arrives at the expected reciprocal saturation charge of the chamber, 1/*M*_*s*_. Following the recommendations of the IAEA CoP TRS-398, determination of 1/*M*_*s*_ using the operating voltage of the chamber and a voltage in the linear region would result in an overestimation of the saturation charge at high collecting voltages^29^. As such, 1/*M*_*s*_ was determined only by using the lowest two collecting voltages of $75$ V and $200$ V. This value was then used to calculate the collecting efficiency of the chamber for a given collecting voltage, $V$, using an equation such as the one below.

8 $${k}_{s,V}=\frac{{M}_{s}}{{M}_{V}}$$

#### Di Martino model

Studies following Boag’s show that, for high dose-per-pulse IORT beams, the standard recombination correction technique could overestimate the recombination factor by $\mathrm{20 \% }$. As such, *Di Martino et al*. *(2005)* developed a dose-per-pulse dependent correction procedure^27^.

9 $${k}_{s,DM}=\frac{\beta {D}_{w,\theta ,eff}}{ln\left(1+\frac{{e}^{p\beta {D}_{w,\theta ,eff}}-1}{p}\right)}$$

where *β* is a factor which is dependent on the recombination coefficient and the calibration coefficient of the chamber and ${D}_{w,\theta ,eff}$ is the effective dose-per-pulse to water, where $\theta$ indicates the dose is per-pulse. This model was derived from Eq.(5), however, defining the initial charge density produced in terms of the number of pulses. They studied the recombination factor of the Roos chamber at $100$ V collecting voltage. The dose-per-pulse range investigated was between $3.97$ cGy and $12.36$ cGy (±2%), giving a ${k}_{s}$ between $1.58$ and $2.34$ (±3.5%). They found that, using the least squares method, their model fitted extremely well to the data, predicting $p=0.179\pm 2.0 \%$.

#### Logistic model

*Petersson et al*.^26^ investigated the recombination correction factor for the Advanced Markus ion chamber, which has a smaller cavity volume than the Roos. The dose-per-pulse investigated ranged from $7$ mGy to $15$ Gy and was determined through relative methods. The TVA technique was shown to only be valid for dose-per-pulse values up-to $10$ mGy. A logistic equation which was used by *Petersson et al*. is shown below.

10 $${k}_{s,L}={\left(1+{\left(\frac{DPP}{V}\right)}^{\alpha }\right)}^{\beta }$$

where *DPP* is the dose-per-pulse in mGy/pulse and $\alpha$ and $\beta$ are fitting constants with no physical meaning.
